# From delay to diagnosis: Chronic invasive fungal rhinosinusitis presenting with facial and orbital complications

**DOI:** 10.1002/ccr3.7600

**Published:** 2023-06-20

**Authors:** Abhinav Suri, Precious Fortes, Benjamin H. Chan, Carolyn J. Sachs

**Affiliations:** ^1^ David Geffen School of Medicine at UCLA Los Angeles California USA; ^2^ Department of Pathology UCLA Health Los Angeles California USA; ^3^ Department of Emergency Medicine UCLA Health Los Angeles California USA

**Keywords:** chronic invasive fungal rhinosinusitis, CIFRS, fungal infections, orbital and facial fungal sinusitis, rhinosinusitis

## Abstract

**Key Clinical Message:**

Early identification and management of chronic invasive fungal rhinosinusitis (CIFRS) is key to optimizing outcomes. A missed diagnosis can result in permanent vision loss, chronic facial pain, or death. We present a case of CIFRS and literature review.

**Abstract:**

This case report presents a 56‐year‐old female with CIFRS involving orbital and facial complications. The patient experienced delayed diagnosis despite multiple ED visits for sinusitis with progressive facial pain and ocular deficits not alleviated with antibiotics, emphasizing the importance of early identification and maintaining high clinical suspicion for CIFRS. Prompt recognition, initiation of antifungal therapy, and aggressive surgical debridement were crucial for preventing disease progression and improving the patient's quality of life.

## Graphical abstract

A 56‐year‐old female presenting with vision loss and facial pain for several months found to have chronic invasive fungal rhinosinusitis with an orbital involvement visualized on MRI and angioinvasive elements identified on biopsy.

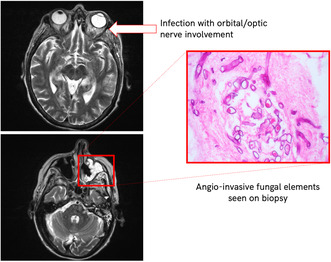

## 1 BACKGROUND

Fungal rhinosinusitis (FRS) is a growing cause of rhinosinusitis across the world.^1^ As a clinical entity, FRS encompasses three different types: acute invasive FRS, granulomatous invasive FRS, and chronic invasive FRS (CIFRS).^2^ While the acute invasive variant has been covered extensively in prior literature and is associated with a high risk of mortality, the chronic invasive category has received less attention but is still equivalently critical to diagnose and treat promptly due to a high mortality rate.^3^ CIFRS is defined as a slowly growing destructive process (>4 weeks) of any paranasal sinus with vascular invasion, inflammatory reaction, and involvement of other local structures.^4^ If left untreated, CIFRS can spread to involve the orbits, resulting in vision loss, and also may grow to involve the brain, carrying high mortality (up to 80%).^5^ Furthermore, CIFRS symptoms can initially present indolently but result in significant deficits to quality of life due to facial numbness, facial drooping, and vision loss.

## 2 OBJECTIVE

Here we present a case of CIFRSwith orbital and ocular involvement. Of note, this patient saw several providers before the diagnosis of FRS was made, highlighting the importance of picking up on exam findings suggestive of CIFRS. We aim to describe unique aspects of this particular case that have not been described in the literature and also highlight initial presenting symptoms are typical of CIFRS along with common predisposing conditions.

## 3 CASE REPORT

The patient is a 56‐year‐old female with history of type II diabetes mellitus (A1c = 9.5%), CKD (Stage 3B), cerebrovascular accident (without residual deficits), and hypertension who presented to the ED for evaluation of a left palate ulcer, left facial pain and paralysis, ocular palsy, and vision loss in her left eye for the past 4 months.

Five months ago, the patient fell asleep on her dentures, causing a hard palate lesion that would not heal. Subsequently, she developed left facial pain, thick nasal discharge, progressive left eye blindness, eye‐opening difficulty, and dysarthria. Despite multiple ED visits and an admission (and administration of antibiotics) for suspected sinusitis, her symptoms persisted. Another ED visit resulted in steroids being given to her for suspicion of multiple sclerosis (MRI ruled out later). In the 2 weeks prior to admission, her left facial numbness and pain worsened, prompting her to be referred to the ED by a dentists she was seeing the day before admission.

**FIGURE 1:**
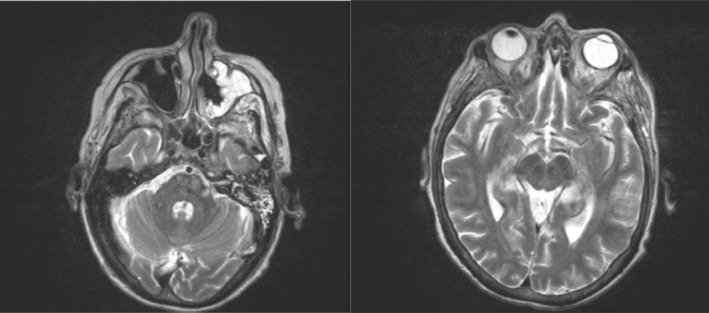
MR brain images. These images shows enhancing inflammatory phlegmon (left image) extending from left maxillary sinus through orbital floor and involving intraconal orbital fat, the optic nerves, optic nerve sheath, and left orbital apex (right image). Additionally, there is an abnormal swelling and enhancement of the extraocular muscles of the left orbit.

On presentation, the patient's vitals were within normal limits. Her physical exam was notable for left periorbital swelling and tenderness to palpation, ptosis of the left eyelid, fixed pupil dilation with total loss of external ocular muscle movement of the left eye, total sensory loss over CN V2 distribution, and a hard palate fistula with erosion into the left maxillary sinus. A detailed ENT examination and biopsy found that there was copious green inspissated crusting in the anterior nasal cavity with evidence of a necrotic inferior and middle turbinate. Frozen sections of the inferior and middle turbinate were taken that (on initial read) did not show fungal elements. Further imaging (shown in Figure 1) showed left maxillary sinusitis with extension into the maxillary sinus walls, palate, infratemporal fossa, and left orbit (with involvement of the optic nerve and extraocular muscles).

**FIGURE 2:**
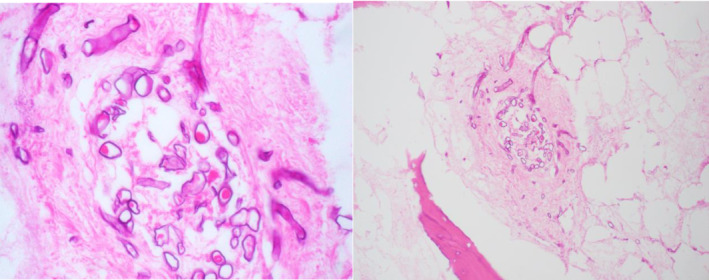
Maxillary sinus hematoxylin and eosin stain (H&E, 60× and 20×) showing broad, aseptate hyphae consistent with invasive fungal elements within necrotic bone and evidence of angioinvasion.

Given the results of exam and biopsy findings of necrotic tissue, the patient was admitted to the hospital for concern of invasive fungal versus bacterial sinusitis. The patient was prophylactically placed on vancomycin and piperacillin–tazobactam, IV amphotericin B, and retrobulbar amphotericin injections per recommendations from ophthalmology. The ENT service proceeded with endoscopic sinus surgery with left medial maxillectomy and left pterygopalatine fossa dissection and debridement and collected several surgical specimen for analysis. Surgical pathology results showed aseptate fungal elements (likely mucormycosis) with evidence of angioinvasion (Figure 2 **)**. Fungal PCR was performed and detected *Blastomyces dermatitidis* or *glichristii*. Additional testing was performed to evaluate Mucorales by PCR. Vancomycin and piperacillin–tazobactam were stopped upon pathology results and isavuconazole was started since the patient developed worsening renal function likely in response to amphotericin.^6^ Patient was additionally started on caspofungin given caspofungin's potential efficacy against invasive fungal infections in combination therapy.^7^ Repeat MR showed no further disease progression or collection.

Over the course of the hospitalization, debridement surgeries, and antifungal treatments, the patient regained some movement of her extraocular muscles and was able to raise her eyelid. Her facial pain greatly improved. She was discharged with ENT follow‐up 26 days after admission.

## 4 DISCUSSION

In this patient, prompt recognition of potential rhino‐orbital fungal sinusitis was key to preventing disease progression, underscoring the importance of identification and management of this clinical entity. Additionally, the clinical history of prior attempts of treatment of sinusitis with antibiotic therapy (without alleviation of symptom progression) and attempts to alleviate symptoms with steroids (which could have worsened progression of her disease) emphasizes the need to keep a chronic fungal etiology high on the differential for a chronic process. Here we discuss more information about the causes of CIFRS and rhino‐orbital fungal infections in general.

### 4.1 Etiology and initial clinical presentation

Aspergillus species are the most common causative agents of invasive rhino‐orbital fungal infections, followed by Mucorales.^8^ While *Aspergillus* species are more commonly seen in immunocompetent patients, Mucorales are often identified in patients with uncontrolled diabetes mellitus and other predisposing factors.^9^

In terms of initial clinical presentation, a retrospective review of 16 CIFRS cases in Beijing Tongren hospital from 2006 to 2014 found that eye pain/other ocular symptoms was the most common chief complaint, with headache being the next most common.^10^ The patient covered in this case study similarly not only had a history of ocular pain/dysfunction, but also had a primary complaint of facial paralysis/pain that prompted her to visit the ED. Immunocompromising conditions such as diabetes were present in a majority of the patients. One study mentioned that among 15 patients diagnosed with CIFRS in the setting of COVID‐19, the average HbA1c was 9.9 ± 2.1 among the subset of individuals who had diabetes.^11^ Similarly, our patient had an HbA1c of 9.5.

### 4.2 Diagnostic workup and treatment

In the cases surveyed, the initial diagnostic workup involved CT and/or MR imaging. Initial CT can show increased density of soft tissue in paranasal sinuses with potential destruction of bones. MR is essential to get next in order to determine extent of soft tissue involvement in the orbital apex and brain parenchyma. Concurrently, biopsy can be attempted to determine whether fungal elements are present or not. However, as in this case, a negative frozen pathological sample does not preclude a diagnosis of CIFRS. Prior studies have shown a false negative rate of frozen samples as high as 16.6% (sensitivity = 0.8337) in intraoperative samples (no studies exist for examining bedside samples as was seen in this case).^12^ PASF‐fs stains have been identified to reduce the false‐negative rate to as low as 5%.^13^

The management of invasive rhino‐orbital fungal infections requires a multidisciplinary approach involving medical and surgical interventions. In terms of medical management, targeting the underlying fungal infection with amphotericin B or isavuconazole was found to be equally effective.^14^ Additionally, early initiation of antifungal therapy and aggressive surgical debridement of infected tissues are crucial for successful outcomes.^15^

In conclusion, this case report highlights the challenges in diagnosing CIFRS with orbital and ocular involvement, underlining the importance of clinical suspicion, thorough examination, and timely intervention. The patient's delayed diagnosis and treatment emphasize the need for increased awareness among healthcare providers to recognize CIFRS as a potentially devastating condition that requires prompt and aggressive management.

## AUTHOR CONTRIBUTIONS

**Abhinav Suri:** Conceptualization; data curation; formal analysis; funding acquisition; investigation; methodology; resources; writing – original draft. **Precious Fortes:** Conceptualization; data curation; visualization; writing – original draft; writing – review and editing. **Benjamin H. Chan:** Conceptualization; data curation; writing – review and editing. **Carolyn J. Sachs:** Conceptualization; data curation; project administration; writing – original draft; writing – review and editing.

## FUNDING INFORMATION

None.

## CONFLICT OF INTEREST STATEMENT

The authors declare no conflicts of interest.

## CONSENT STATEMENT

Written informed consent was obtained from the patient to publish this report in accordance with the journal's patient consent policy.

## Data Availability

Data available on request due to privacy/ethical restrictions.
